# The *Treponema pallidum* outer membrane protein repertoire and the quest for a syphilis vaccine

**DOI:** 10.5327/dst-2177-8264-1480

**Published:** 2026-01-19

**Authors:** Everton Burlamarque Bettin, Andre Alex Grassmann, Kelly Lynn Hawley, Melissa Jo Caimano, Justin David Radolf

**Affiliations:** 1 UConn Health, Department of Medicine – Farmington, Connecticut, USA.; 2 Connecticut Children’s Research Institute – Hartford, Connecticut, USA.

**Keywords:** *Treponema*, Syphilis, OMPeome, Vaccine, Extracellular loops, Scaffolds, *Treponema*, Sífilis, OMPeoma, Vacina, Alças extracelulares, Scaffolds

## Abstract

Despite more than a century of investigation, syphilis vaccine development has long been hindered by the unusual outer membrane of *Treponema pallidum* subsp. *pallidum* (*TPA*) and the historical inability to propagate the spirochete *in vitro*. Early observations using the rabbit model established that protective, antibody-mediated immunity is achievable. The recent characterization of the repertoire of *TPA* outer membrane proteins (OMPs) defined the universe of potential targets for protective antibodies and provided a critical foundation for ongoing syphilis vaccine development. Built on a “learning from nature” approach, the mapping of antibody responses elicited during natural infection against OMPs allowed the identification and prioritization of extracellular loops as vaccine targets. Immunization of animals with protein scaffolds displaying these targets generates high titers of antibodies able to recognize surface-exposed regions of the spirochete. Recent advances in long-term *in vitro* cultivation and genetic manipulation of *TPA* have enabled the development of assays to directly evaluate the functional activity of extracellular loops — specific antibodies in promoting opsonophagocytosis, growth inhibition, impairment of motility, and outer membrane disruption. Next-generation platforms are being explored to enhance immunogenicity, simplify production, and facilitate scalable translation of these immunogens toward clinical evaluation. In parallel, researchers are uncovering the sequence variability in OMPs across circulating *TPA* strains to understand how mutations can affect antibody recognition and global vaccine efficacy. Collectively, these advances position the field to leverage structural, immunological, and microbiological insights to counter the stealth pathogen and, ultimately, achieve an effective syphilis vaccine.

## RESUMO

Apesar de mais de um século de investigação, o desenvolvimento de uma vacina contra a sífilis tem sido dificultado pela membrana externa incomum de *Treponema pallidum* subsp. *pallidum* (*TPA*) e pela incapacidade histórica de propagar a espiroqueta *in vitro*. Observações iniciais utilizando o modelo animal coelho demonstraram que uma imunidade protetora mediada por anticorpos é possível. A recente descoberta do repertório de proteínas de membrana externa (OMPs, *outer membrane proteins*) do *TPA* definiu os possíveis alvos de anticorpos protetores e proporcionou a base crítica para o desenvolvimento atual de vacinas contra a sífilis. Baseando-se em uma abordagem denominada “*learning from nature*” (aprendendo com a natureza), o mapeamento dos anticorpos induzidos durante a infecção natural contra as OMPs permitiu a identificação e priorização de alças extracelulares como alvos vacinais. A imunização de animais com *scaffolds* (esqueletos) proteicos que exibem esses alvos gera títulos elevados de anticorpos capazes de reconhecer regiões expostas na superfície da espiroqueta. Avanços recentes no cultivo *in vitro* de longo prazo e na manipulação genética do *TPA* possibilitaram o desenvolvimento de ensaios para avaliar diretamente a atividade funcional das alças extracelulares — anticorpos específicos na promoção da opsonofagocitose, inibição do crescimento, comprometimento da motilidade e ruptura da membrana externa. Plataformas de segunda geração estão sendo exploradas para aumentar a imunogenicidade, simplificar a produção e facilitar a escalabilidade desses imunógenos até ensaios clínicos. Paralelamente, pesquisadores estão identificando a variabilidade da sequência de OMPs entre cepas circulantes do *TPA* para compreender como mutações podem afetar a ligação de anticorpos e a eficácia global da vacina. Coletivamente, esses avanços posicionam estrategicamente o campo para alavancar conhecimentos estruturais, imunológicos e microbiológicos, visando, finalmente, alcançar uma vacina eficaz contra a sífilis.

## INTRODUCTION

Syphilis has been recognized as one of the most feared sexually transmitted diseases since the late 15th century, with devastating clinical manifestations and enduring social stigma^(1)^. The modern era of investigation into this enigmatic disease began in 1906 with the identification of its causative agent, the spirochetal pathogen *Treponema pallidum* subsp. *pallidum* (*TPA*), by Schaudinn and Hoffmann^(2)^. Despite more than a century of research, the development of a syphilis vaccine has lagged behind other bacterial pathogens, largely due to the unusual outer membrane biology of *TPA*^(3)^ and the historical inability to propagate syphilis spirochetes *in vitro*. Nevertheless, early observations established that protective immunity against *TPA* is achievable^(4,5)^. This concept was strengthened by a landmark 1948 study by Magnuson and Rosenau^(6,7)^, which demonstrated that rabbits infected with *TPA* for increasing lengths of time prior to penicillin treatment required progressively higher challenge inocula to develop symptomatic reinfection.

Although these findings provided compelling evidence for acquired immunity, the immunological basis of protection remained unclear. Subsequent studies pointing to antibodies showed that pre-incubation of *TPA* with immune rabbit sera (IRS)^(8)^ or human syphilitic sera (HSS)^(9)^ reduced lesion formation in rabbits, providing the first direct evidence that protective factors resided in immune sera. Passive immunization experiments later confirmed antibodies as central mediators of host defense against *TPA*^(10,11)^. The mechanism by which these functional antibodies conferred protection was ultimately clarified through studies using rabbit peritoneal macrophages, which identified opsonophagocytosis as a primary pathway for spirochete clearance^(12)^. Together, these observations provided proof that protective antibodies must recognize antigens exposed on the spirochete surface — an insight that continues to guide contemporary syphilis vaccine development. The ability of antibodies to serve as mediators of protective immunity is in accord with evidence that the syphilis spirochete is an extracellular bacterium^(13)^.

### The *Treponema pallidum* outer membrane protein repertoire and the quest for syphilis vaccine candidates

With evidence in hand that functional antibodies can mediate protection, the syphilis vaccine field was confronted with a central question: which surface-exposed antigens of *TPA* serve as targets of protective immunity? Identifying such antigens proved unusually challenging due to the atypical ultrastructure of the *TPA* outer membrane (OM), a defining feature of the bacterium that led to its designation as a “stealth pathogen”^(3,14)^. Unlike most diderm bacteria, *TPA* lacks the highly antigenic lipopolysaccharide and, instead, possesses an OM in which surface-exposed outer membrane proteins (OMPs) are present at exceptionally low density, resulting in poor immunogenicity^(15)^. The intrinsic fragility of the *TPA* OM further complicated early efforts to identify surface-exposed targets. Routine sample preparation often leads to disruption of the spirochete’s OM, producing artifactual surface labeling and erroneous conclusions about surface exposure that have shaped decades of unsuccessful vaccine strategies^(3,16)^. Subsequent microscopy and immunofluorescence analyses using antisera against a panel of recombinant, highly antigenic lipoproteins (e.g., Tpp15/TP0171, Tpp17/TP0435, TP0971/Tpp34, and Tpp47/TP0574) demonstrated that these immunogens are located beneath the OM (i.e., within the periplasm) rather than exposed on the spirochete surface^(17,18)^. These observations reinforced the emerging view that only integral OMPs are antibody-accessible and, therefore, *bona fide* targets of functional antibodies.

**Figure 1.**
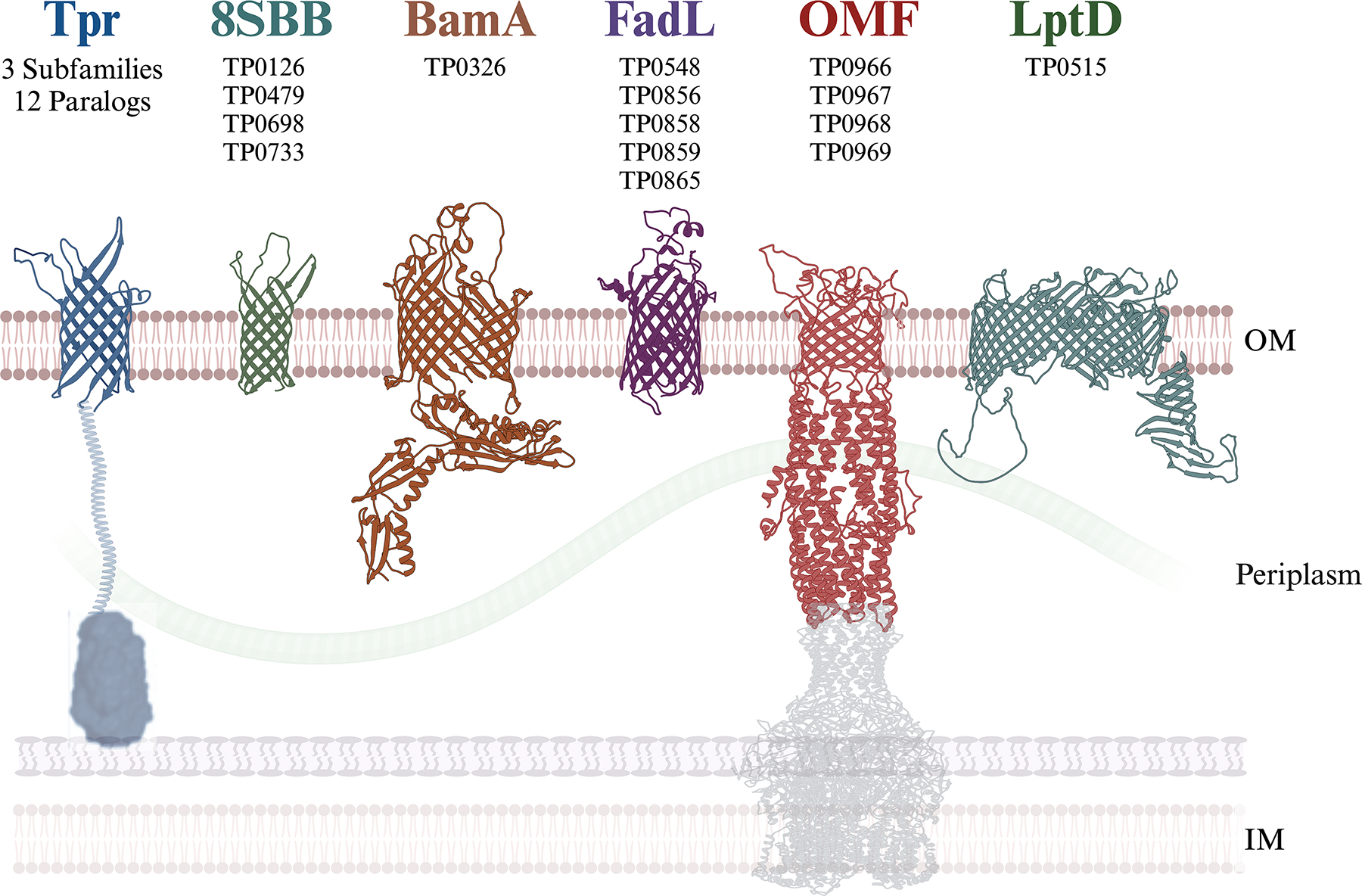
The Treponema pallidum outer membrane protein repertoire. Tpr: *Treponema pallidum* repeat proteins; 8SBB: 8-stranded β-barrels; BamA: β-barrel assembly machinery A; FadL: long-chain fatty acid transporters orthologs; OMF: outer membrane factor for efflux pumps; LptD: lipopolysaccharide transport protein D; OM: outer membrane; IM: inner membrane.

The completion of the *TPA* genome in 1998^(19,20)^ appeared to offer a straightforward path for the systematic identification of *TPA* OMPs. However, early genome-mining efforts failed to identify candidates with sequence homology to canonical Gram-negative OMPs^(3)^. After years of largely unproductive searches for sequence-based orthologs, a conceptual shift in the field occurred when our group adopted a structure-based strategy focused instead on the identification of proteins predicted to form β-barrels^(21)^, a defining feature of transmembrane OMPs^(22)^. This approach enabled the identification of the spirochete’s OMP repertoire (OMPeome) and provided a critical foundation for the ongoing syphilis vaccine development. More recently, advances in protein structure prediction and machine-based learning approaches have further expanded the OMPeome^(23)^, revealing that the *TPA* OM, while sparsely populated, is more complex and Gram-negative-like than originally appreciated. The currently known *TPA* OMPeome includes two stand-alone proteins, β-barrel assembly machinery A (BamA) and lipopolysaccharide transport protein D (LptD), both involved in OM biogenesis, and four paralogous families involved in nutrient uptake or extrusion of noxious substances across the OM: 8-stranded β-barrels (8SBB), OM factors for efflux pumps, *TPA* repeat proteins (Tpr), and orthologs for long-chain fatty acid transporters (FadL) (Figure 1)^(23)^.

### Learning from nature to prioritize vaccine targets

The definition of the *TPA* OMPeome established the potential targets for functional antibodies responsible for protection against reinfection. Mapping the natural antibody responses to OMPs elicited during infection, underpinning a “learning from nature” strategy, provided a rational framework for prioritizing vaccine candidates^(24,25)^. To characterize this response, IRS and HSS were interrogated against identified *TPA* OMPs. However, full-length recombinant OMPs are notoriously difficult to express in heterologous systems (e.g., *Escherichia coli*) and often fail to adopt or maintain their native conformations outside the context of the OM^(26)^. Because proper folding and preservation of conformational epitopes are essential for accurately assessing antibody recognition, this limitation required alternative experimental strategies. Although OMPs are the surface-exposed targets of *TPA*, the majority of each protein is embedded within the membrane, with only discrete extracellular loops (ECLs) accessible to antibody binding^(27)^. Recent advances in protein structural prediction^(28,29)^ have enabled precise mapping of these ECLs. Guided by these structural predictions, our group developed a structure-based approach in which individual *TPA* ECLs were displayed on a *Pyrococcus furiosus* thioredoxin (*Pf*Trx) scaffold^(30)^ to generate soluble, conformationally constrained ECL antigens. This approach circumvented the technical challenges associated with full-length OMPs and enabled systematic identification of accessible, immunologically relevant regions within *TPA* OMPs^(24,25)^. Using this platform, we have identified multiple ECLs that are immunogenic during both rabbit and human infection, providing direct evidence that the host immune system can, and does, recognize these rare surface-exposed targets during natural infection^(25,31–33)^. Together, these findings support the rational prioritization of surface-exposed targets for syphilis vaccine development.

### Current strategies to develop a syphilis vaccine

Building on the “learning from nature” conceptual framework, another central question is whether immunogenic ECLs can elicit functional, protective antibody responses upon immunization. Along these lines, immunogenic ECLs from BamA and three FadL orthologs (TP0856, TP0858, and TP0865) served as our initial targets for immunization studies. As expected, immunization of rabbits and mice with individual *Pf*Trx-ECL constructs elicited high titers of ECL-specific IgG, confirming that these regions retain immunogenicity upon artificial immunization when displayed on a scaffold^(31,34)^. However, immunogenicity alone is insufficient; to qualify as effective vaccine antigens, the elicited antibodies must promote spirochete clearance. Recent advances in long-term *in vitro* cultivation^(35)^ and genetic manipulation of *TPA* enabled our group to systematically evaluate sera obtained from immunized animals across multiple functional assays. These studies revealed that ECL-directed antibodies contribute to *TPA* clearance through complementary mechanisms. Sera from animals immunized with *Pf*Trx-ECLs promoted opsonophagocytosis of *TPA* by both rabbit peritoneal macrophages and murine bone marrow–derived macrophages at levels comparable to those observed with immune serum generated during infection^(34)^. In addition to enhancing phagocytic uptake, ECL-specific sera impaired multiple aspects of spirochete biology *in vitro*, including reductions in viability, motility, and host-cell attachment^(13,33,34)^. Our findings further indicated that ECL-specific antibodies may interfere with OMP function, ultimately compromising the integrity of the fragile *TPA* OM. To directly assess this possibility, we engineered a *TPA* strain constitutively expressing green fluorescent protein^(13)^. Using a flow cytometry-based assay, we observed that incubation of *TPA* with *Pf*Trx-ECL antisera promoted OM disruption and dose-dependent growth inhibition *in vitro*, providing direct evidence that surface-directed antibodies can exert bactericidal effects against the spirochete^(13,33)^. Importantly, these findings were not limited to *in vitro* assays; intradermal challenge of rabbits using treponemes pre-incubated with growth-inhibitory ECL-specific antibodies resulted in absent or transient lesions with substantially lower bacterial burdens^(33)^. Although a formal correlate of protection for syphilis has yet to be established, the combined readouts of opsonophagocytosis, growth inhibition, impairment of motility, OM disruption, and neutralization of infectivity now provide quantitative functional surrogates previously lacking in the field, enabling a rational evaluation of vaccine candidates to be prioritized^(25)^.

While recombinant *Pf*Trx-ECL immunogens establish proof of concept, next-generation platforms are also being explored to enhance immunogenicity, simplify production, and facilitate scalability toward clinical evaluation. We recently developed messenger ribonucleic acid (mRNA)-based immunogens encoding *Pf*Trx-*TPA* ECLs previously shown to elicit functional antibodies as recombinant proteins (unpublished data). These constructs are efficiently translated *in vivo* and induce antibody responses upon immunization, representing a notable achievement in vaccine development for syphilis. In parallel, we generated virus-like particle (VLP)-based immunogens (unpublished data), which provide highly ordered, repetitive antigen display and are well known to elicit potent humoral and cellular immune responses^(36,37)^. Both platforms can stimulate strong immunity even in the absence of external adjuvants, simplifying the formulation, storage, and manufacturing relative to recombinant protein–based approaches, while also supporting rapid and scalable production^(37,38)^.

The low antigenic density of the *TPA* surface and interindividual variability in immune responses suggest that an effective syphilis vaccine will likely require a multivalent strategy. Scaffolds capable of simultaneously displaying multiple ECLs therefore represent a logical next step, enabling presentation of multiple protective epitopes by a single immunogen. To this end, our group has designed multivalent scaffolds accommodating up to four ECLs, including the C-lobe of *Neisseria meningitidis* transferrin-binding protein B (TbpB)^(39,40)^ and a truncated form of *Escherichia coli* outer membrane protein A (OmpAtr)^(41)^. These constructs elicit robust antibody responses against multiple ECLs without compromising reactivity to individual components, achieving titers comparable to those induced by single-ECL *Pf*Trx constructs^(42)^.

### Insights from the global *Treponema pallidum* outer membrane protein repertoire

Collectively, these complementary strategies highlight promising paths toward a syphilis vaccine capable of achieving broad, global protection. Reaching this goal, however, requires explicit consideration of the sequence variability present in vaccine targets across circulating *TPA* strains worldwide, as even single amino acid substitutions can alter antibody recognition and compromise vaccine efficacy^(43,44)^. Analysis of sequence variability is particularly critical for immunogenic, surface-exposed ECLs, where strong antibody binding is expected to impose immune pressure and promote the emergence of escape variants^(45)^. Our group and others have sequenced clinical *TPA* strains from multiple geographic regions to define the global *TPA* OMPeome, the complete set of OMP variants across circulating strains^(46–50)^. These analyses revealed sequence variability within OMPs in regions predicted to be extracellular and harboring B cell epitopes, suggesting that host immune pressure is a major driver of OMP diversity^(46,47)^. Notably, and somewhat surprisingly, many vaccine-relevant ECLs that elicit functional antibodies upon immunization (e.g., BamA ECL4, TP0856 ECL2, and ECL4) remain highly conserved within clinical strains^(46,47)^. The persistence of sequence invariance in these ECLs, despite their immunogenicity, raises intriguing questions regarding the nature and magnitude of immune pressure acting on them during natural infection. One possibility is that strong functional or structural constraints limit protein diversification without compromising essential OMP function^(47)^. Alternatively, antibodies generated during natural infection may be quantitatively or qualitatively insufficient to exert meaningful selective pressure. These observations underscore an additional layer of complexity in vaccine design, highlighting potential differences between infection- and vaccine-induced antibody responses. For protein regions with more variability (e.g., TP0858 ECL4, TP0865 ECL3), our identification of alternative proteoforms circulating within distinct clinical lineages enables prediction of how naturally occurring mutations may influence antibody recognition of “wild-type” epitopes^(47,51)^. Incorporating these insights refines antigen prioritization and supports rational vaccine design strategies that explicitly account for global sequence diversity within the *TPA* OMPeome. Ultimately, understanding how immune and evolutionary pressures shape OMP variation in *TPA* is essential for identifying vaccine targets that are both biologically indispensable and capable of eliciting durable, protective immunity.

## CONCLUSION

**Figure 2.**
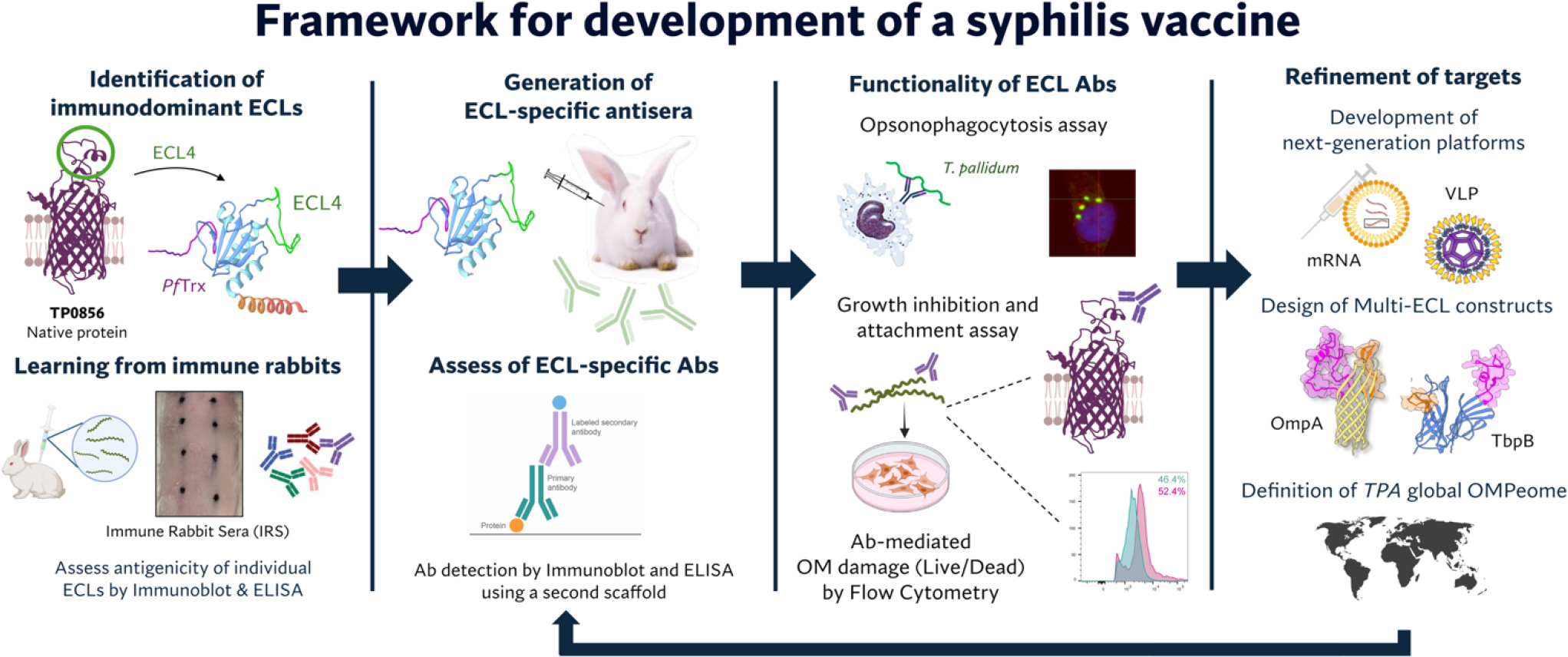
Framework for development of a syphilis vaccine. ECLs: extracellular loops; P*f*Trx: *Pyrococcus furiosus* thioredoxin; ELISA: Enzyme-linked immunosorbent assay; Abs: antibodies; mRNA: messenger ribonucleic acid; VLP: virus-like particle; OmpA: outer membrane protein A; TbpB: transferrin-binding protein B.

The past decade has marked a turning point in syphilis vaccine research, shifting the field from questioning whether a vaccine is achievable to defining how one can be designed rationally. Rapid and coordinated advances in structural biology, immunology, and microbiology have reshaped our understanding of *TPA*. The growing evidence that immunization can elicit functional antibodies against surface-exposed targets has provided a mechanistic framework for comparing immunogens and prioritizing those with the greatest potential to elicit protective immunity (Figure 2). Although current functional readouts capture key mechanistic properties of antibody activity against *TPA*, they represent surrogate measures rather than definitive predictors of protection. Establishing true correlates of protection will require directly linking antibody functionality to protection outcomes in well-controlled challenge studies. In this regard, reductions in lesion development and treponemal dissemination following rabbit infection remain the gold-standard endpoints for evaluating protective immunity. Expanding these analyses through the development and refinement of additional animal models, including murine systems^(52,53)^, will further facilitate systematic evaluation of immune responses and vaccine efficacy^(34,54)^. Establishing robust correlates of protection will be critical for translating promising immunogens into clinical trials, as such benchmarks are required to predict protective immunity in humans^(33)^. Recent progress indicates that the remaining barriers to syphilis vaccine development are no longer conceptual but technical. Over the coming years, the field is poised to integrate rationally designed antigens, quantitative immunological metrics, and scalable translational platforms to advance the most promising candidates from bench to clinic. With this foundation in place, syphilis vaccine research is now well-positioned to counter the stealth pathogen and, ultimately, achieve a safe and effective vaccine for a disease that has inflicted untold misery upon humankind for centuries^(1)^.
